# Metagenomic Analysis Reveals New Microbiota Related to Fiber Digestion in Pigs

**DOI:** 10.3389/fmicb.2021.746717

**Published:** 2021-11-18

**Authors:** Gensheng Liu, Pinghua Li, Liming Hou, Qing Niu, Guang Pu, Binbin Wang, Taoran Du, Sung Woo Kim, Peipei Niu, Qiang Li, Ruihua Huang

**Affiliations:** ^1^Institute of Swine Science, Nanjing Agricultural University, Nanjing, China; ^2^Huaian Academy of Nanjing Agricultural University, Huaian, China; ^3^Institute of Animal Husbandry and Veterinary Science, Shanghai Academy of Agricultural Sciences, Shanghai, China; ^4^Department of Animal Science, North Carolina State University, Raleigh, NC, United States; ^5^Huaiyin Xinhuai Pig Breeding Farm, Huaian, China

**Keywords:** pig, apparent fiber digestibility, gut microbiota, shotgun metagenomic sequencing, high fiber coproducts

## Abstract

Making full use of high fiber and low-cost crop coproducts is helpful to alleviate the situation of people and livestock competing for crops. Digestion of dietary fibers in pigs is mainly through microbial fermentation in the large intestine. To reveal microbiota related to fiber digestion in pigs, fecal samples have been collected from 274 healthy female Suhuai pigs at 160 days of age under the same feeding conditions and have measured apparent neutral detergent fiber (NDF) and acid detergent fiber (ADF) digestibility. Samples from Suhuai pigs with extreme high and low apparent NDF digestibility and extreme high and low apparent ADF digestibility were subjected to shotgun metagenomic sequencing. At the species level, 62 microbial species in H_NDF group and 54 microbial species in H_ADF group were related to high fiber digestibility. Among them, *Lachnospiraceae bacterium 3-1* and *Alistipes* sp. *CAG:514* may be new types of microorganisms associated with fiber digestion. In addition, we found that more abundant GH5 and GH48 family (contribute to cellulose degradation) genes, GH39 and GH53 family (contribute to hemicellulose degradation) genes in microorganisms may contribute to the higher apparent NDF digestibility of pigs, and more abundant GH3 and GH9 family (contribute to cellulose degradation) genes in microorganisms may contribute to the higher apparent ADF digestibility of pigs. The abundance of AA4 family (helps in lignin degradation) genes in H_NDF and H_ADF groups was significantly higher than that in L_NDF and L_ADF groups, respectively (*P* < 0.05). Three pathways in H_NDF group and four pathways in H_ADF group are important pathways associated with degradation of non-starch polysaccharides, and their relative abundance is significantly higher than that in L_NDF and L_ADF groups, respectively. Gut microbiota of Suhuai pigs with high apparent fiber digestibility had higher abundance of genes and microbiota related to fiber digestion and may have stronger fiber digestion potential compared with low apparent fiber digestibility group. This study revealed that the characteristics of gut microbiota and microbial gene functions of pigs with high fiber apparent digestibility, which provided a theoretical basis and reference for further understanding the impact of gut microbiota on fiber digestibility of pigs.

## Introduction

Corn and soybean meal are the main components of high energy and high protein diets for pigs and are also the main raw materials of food products for human consumption, fermentation, and bioenergy industry (Sevillano et al., 2018). As the arable land of food crops was limited whereas the world’s population continues to increase, the feed industry is being restricted. Feed suppliers are unable to get enough feed raw materials, which leads to the decline of feed output and the rise of feed prices (Kim et al., 2019). Feed cost accounts for about 70% of the cost of pig farming, and the rise of feed prices has seriously restricted the production of pork products.

High feed prices force us to find and use alternative coproducts. Non-conventional feedstuffs (NCFs) are coproducts of crop processing, which have the characteristics of high fiber content and low cost. The use of NCF is an effective measure to solve the shortage of feed raw materials in the feed industry and helps to reduce the cost of livestock products (Negesse et al., 2009). Therefore, how to make full use of agricultural by-products in pig industry is particularly important. This is conducive to improve the income of raising pigs and alleviate the competition for grain between people and animals. At present, in order to make full use of locally grown feedstuffs and reduce the cost of raising pigs, a variety of fibrous coproducts, such as wheat bran, rice bran, and soybean bran, are added to the pig diet (Agyekum and Nyachoti, 2017).

The main composition of dietary fiber is carbohydrate, which is usually classified into two categories according to its water solubility: insoluble dietary fiber (IDF), including cellulose part of hemicelluloses, and lignin; and soluble dietary fiber (SDF), including pentosans, pectin, gums, and mucilage (Maćkowiak et al., 2016; Leon, 2019; Williams et al., 2019). At present, there are many methods to analyze the fiber content in animal feed. The determination method of neutral detergent fiber (NDF) and acid detergent fiber (ADF) in feeds (Waldern, 1971) has been widely used in the world. NDF in feeds mainly contains cellulose, hemicelluloses, and lignin; ADF in feeds mainly contains cellulose and lignin.

It is notable that as a mammal, the genome of pigs does not encode enzymes that can degrade dietary fiber, and its digestion of dietary fiber is mainly through the fermentation of microorganisms in the large intestine (Flint et al., 2012). Many studies have shown that the microbial taxonomic diversity in the gastrointestinal tract of pigs is enormous and the microbiota may provide numerous biological activities that the host lacks (Backhed, 2005; Isaacson and Kim, 2012). The digestive enzymes secreted by the gastrointestinal tract of pig cannot digest dietary fiber, but the microbiota in the large intestine can encode abundant carbohydrate enzymes, including cellulase, hemicellulase, pectinase, and so on (Varel, 1987; Jones, 2014; Zeng et al., 2019). Some dietary fiber is degraded into oligosaccharides or monosaccharides by fiber degrading enzymes secreted by intestinal microorganisms (Broekaert et al., 2011). Oligosaccharides and monosaccharides are further fermented to produce short-chain fatty acids (SCFAs) such as acetic acid, propionic acid and butyric acid for absorption and utilization by large intestine epithelial cells, which greatly improves the feed utilization rate (Bergman, 1990; Lindberg, 2014). SCFAs produced by microbial fermentation in colon can provide up to 5–20% of pig total energy (Tilg and Kaser, 2011; Ashida et al., 2012), increase the weight of empty gastrointestinal tract (all segments except stomach; Kass et al., 1980) and the oxidative metabolism capacity of the intestine (Weber and Kerr, 2012). Niu et al. (2015) and Adhikari et al. (2019) found that in the gut microbiota of pigs, Firmicutes was dominant, followed by Bacteroides. Duarte et al. (2021) found that adding appropriate amount of β-glucanase and xylanase to pig feed can regulate the mucosa associated microbiota by increasing the relative abundance of beneficial bacteria and reducing potential harmful bacteria. *Fibrobacter succinogenes* (*intestinalis*), *Ruminococcus albus*, *Ruminococcus flavefaciens*, *Butyrivibrio* spp., *Clostridium leptum*, *Prevotella ruminicola*, and *Clostridium herbivorans* are the bacteria with high fiber degradation activity in pig intestines, which can secrete abundant fiber degrading enzymes to help the body digest the dietary fiber that is difficult to degrade (Varel et al., 1995; Varel and Yen, 1997). *Bacteroides succinogenes* is a cellulose degrading bacteria isolated from the pig’s large intestine, and its number will increase after feeding high fiber feed (Varel et al., 1984). Many studies have shown that the cellulolytic bacteria found in pig intestine are similar to those found in rumen. At present, there are still a large number of undiscovered cellulolytic bacteria in pig intestines, and their potential functional relationship is still unclear. Therefore, it is necessary to further study the microbial mechanism of fiber degradation and identify the microorganisms related to fiber digestion, which is of great value to strengthen the application of coproducts in pig industry.

Suhuai pig is a synthetic Chinese breed that bred by crossing Xinhuai pig and Large white Xinhuai pig bred by crossing Huai pig (50%) of Chinese indigenous pig and Large white (50%). It is well-known that Chinese local pig breeds have strong ability of fiber degradation. Suhuai pig containing 25% Chinese indigenous Huai pig ancestry, and many studies have proved that Suhuai pigs have strong fiber digestion ability (Hao et al., 2016; Pu et al., 2020). Suhuai pig is a suitable alternative as an experimental animal to study the efficient utilization of fiber by pig gut microbiota. In previous studies, we collected the feed and fecal samples of 274 healthy Suhuai female finishing pigs aged 160 days and determined the apparent digestibility of NDF and ADF. We found that the coefficients of variation (CV) of apparent NDF and ADF digestibility of Suhuai pigs was 12.08 and 18.08%, respectively (Niu et al., 2020). To detect the characteristics of gut microbiota and microbial gene functions of pigs with high and low fiber apparent digestibility and to reveal microbiota related to fiber digestion in pigs, samples from Suhuai pigs with extreme high and low apparent NDF digestibility and extreme high and low apparent ADF digestibility were subjected to shotgun metagenomic sequencing in this study.

## Materials and Methods

### Animals and Sample Collection

The data of apparent NDF and ADF digestibility were obtained from our previous research (Niu et al., 2020). In the previous studies, we selected 274 healthy Suhuai female finishing pigs aged 160 days with the same feeding conditions. No antibiotics were fed 1 month before sample collection and no obvious disease was found 2 weeks before sample collection. Approximately 200 g of diet samples and fecal samples for the analysis of apparent nutrient digestibility were collected in plastic bags and mixed with 15 mL 10% sulfuric acid on site. In addition, fecal samples from each pig were collected in 2 mL centrifuge tube without any treatment for shotgun metagenomic sequencing. After collection, all samples were stored and transported in ice box for a short time, and then stored at −80°C in ultra-low temperature freezer of laboratory. The diet samples and fecal samples were dried at 65°C in a forced-air oven for 48 h to a constant weight. About 0.5 g of samples were taken and the contents of NDF and ADF in diet sample and each pig fecal sample were determined by ANKOM A200 filter bag technique (Van Soest et al., 1991) and the apparent digestibility of each pig was calculated. The following equation was used to calculate the digestibility of 274 sample (Niu et al., 2020):


(1) $$CAD_{D}\left(\%\right)=100\times \left(1-\frac{D\,C_{F\times A\,I\,A_{D}}}{D\,C_{D\times A\,I\,A_{F}}}\right)$$

where CAD_*D*_ represents the apparent dietary components digestibility; DC_*F*_ represents the dietary component in feces; AIA_*D*_ represents the AIA concentration in diet; DC_*D*_ represents the dietary component in diet; and AIA_*F*_ represents the AIA concentration in feces.

In this study, we selected 5 pigs with the highest apparent NDF digestibility (H_NDF group; average apparent NDF digestibility was 83.30%; SE 5.22), 6 pigs with the highest apparent ADF digestibility (H_ADF group; average apparent ADF digestibility was 75.69%; SE 4.75), 6 pigs with the lowest apparent NDF digestibility (L_NDF group; average apparent NDF digestibility was 53.10%; SE 5.07), and 6 pigs with the lowest apparent ADF digestibility (L_ADF group; average apparent ADF digestibility was 41.93%; SE 8.47) for shotgun metagenomic sequencing to detect the characteristics of gut microbiota and microbial gene functions of pigs with high and low fiber apparent digestibility and to reveal microbiota related to fiber digestion in pigs. Since 3 pigs in L_NDF group and L_ADF group were the same, a total of 20 experimental pigs were performed the metagenomic sequencing.

### DNA Extraction, Library Construction, and Shotgun Metagenomic Sequencing

About 0.2 g feces from each pig were used for shotgun metagenomic sequencing. Fecal DNA was extracted with OMEGA Kit (E.Z.N.A^®^Soil DNA Kit) and transferred to Majorbio (Shanghai, China), whose quality was checked according to manufacturer’s instructions (Wang et al., 2016) for further analysis. Covaris M220 (Gene Company Limited, China) was used to break the extracted DNA into an average size of about 400 bp, and then NEXTFLEX Rapid DNA-Seq (Bioo Scientific, Austin, TX, United States) was used to construct paired-end PE library. The full complement of sequencing primer hybridization sites of adapter was ligated to the blunt-end of fragments. Paired-end sequencing was performed on Illumina NovaSeq (Illumina Inc., San Diego, CA, United States) at Majorbio Bio-Pharm Technology Co., Ltd. (Shanghai, China) using NovaSeq Reagent Kits in accordance with the manufacturer’s instructions.^1^ The sequence data associated with this project has been deposited in the NCBI Short Read Archive database (Accession Number: PRJNA735412).

The raw sequencing reads were performed quality control. Then reads were aligned with the pig genome by BWA^2^ to eliminate any hits associated with the reads and their mated reads. Metagenomics data were mixed and assembled using MEGAHIT (Li et al., 2015).^3^ Contigs with the length being or over 300 bp were retained. MetaGene (Noguchi et al., 2006)^4^ was used to predict the open reading frames (ORFs), and then the ORFs with length being or over 100 bp were retrieved by using the NCBI translation table^5^ and translated into amino acid sequences. All predicted genes were clustered (95% identity, 90% coverage) using CD-HIT (Fu et al., 2012).^6^ The longest sequence in each cluster was selected as representative sequences for constructing non-redundant gene catalog. Clean reads were mapped to the representative sequences (95% identity) using SOAPaligner (Li et al., 2008),^7^ and the abundance of all gene of samples was evaluated.

NCBI NR database was used to obtain the taxonomy annotation and KEGG database (Kyoto Encyclopedia of Genes and Genomes)^8^ was used to predict gene function by BLASTP (BLAST Version 2.2.28+).^9^ Carbohydrate-active enzymes (CAZyme) database (Version 5.0)^10^ was used to annotate carbohydrate-active enzymes by hmmscan.^11^ Relative abundance estimates were based on reads per kilobase per million mapped reads (RPKM) values of metagenomic reads (Lawson et al., 2017).

### Bioinformatics Analysis

The apparent digestibility of NDF and ADF of all subjects was calculated by SAS 9.4 software. R was used for all statistical analysis in this study. Linear discriminate analysis effect size (LEfSe) was used to identify potential biomarkers related to fiber degradation (Segata et al., 2011). Principal coordinates analysis (PCoA) was used to make 2D principal coordinate analysis plots to analyze the differences of microbial community structure in different samples (Vázquez-Baeza et al., 2013). We found an outlier sample in H_NDF group through non-metric multidimensional scaling (NMDS) (Supplementary Figure 2A) and PCoA (Supplementary Figure 2B) based on previous research (in total 21 samples). In order to ensure the accuracy of the experimental results, we removed this abnormal sample for further analysis.

## Results

### Metagenomic Sequencing Data of 20 Stool Samples

A total of 20 experimental pigs with extreme high and low apparent NDF digestibility and extreme high and low apparent ADF digestibility were performed the metagenomic sequencing. A total of 1,890,237,030 raw reads were obtained, each with a length of 150 bp. After quality control and pig genome decontamination, a total of 1,874,777,776 high quality clean reads were acquired, with an average of 93,738,889 high quality clean reads per sample. There are 15,477,706 contigs. The number of open reading frames (ORFs) was 24,613,063 after prediction (Supplementary Table 1). A total of 10,078,965 genes were classified into a non-redundant gene catalog for further analysis, with an average length of 572 bp.

### Microbiota Composition of Neutral Detergent Fiber and Acid Detergent Fiber Groups

**FIGURE 1:**
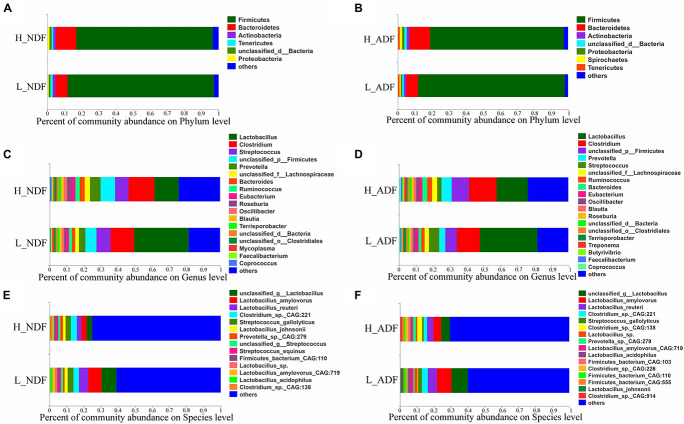
The gut microbiota composition of Suhuai pigs at phylum, genera, and species level. Relative abundance of dominant microbiota at the phylum **(A,B)**, genus **(C,D)**, and species **(E,F)** levels in NDF and ADF groups were shown by stacked bar graphs. Those less than 1% of bacteria were merged into others.

**FIGURE 2:**
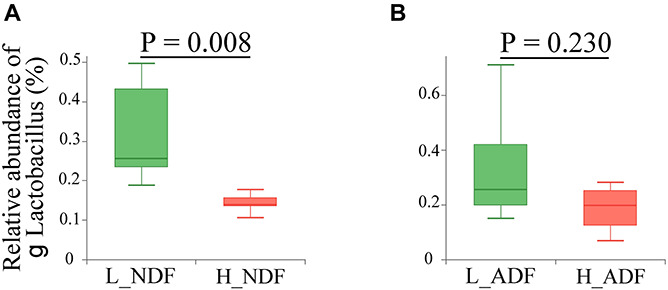
Comparison of the relative abundance of genus *Lactobacillus* between high and low apparent fiber digestibility. The relative abundance difference of genus *Lactobacillus* were compared between H_NDF and L_NDF groups (**A**, *P* < 0.01), and H_ADF and L_ADF groups (**B**, *P* > 0.05) by Wilcoxon rank-sum test, respectively.

At the phylum level, the sum of relative abundance of Firmicutes and Bacteroidetes reached more than 90%, indicating that the microorganisms in Suhuai pig feces are mainly composed of Firmicutes and Bacteroidetes (Figures 1A,B). This result is consistent with previous results of 16S rRNA gene sequencing (Niu et al., 2020). Other microorganisms mainly belong to Actinobacteria, Tenericutes, and Proteobacteria. In NDF and ADF groups, Bacteroidetes accounted for higher proportion in the high digestibility group but it did not reach statistical significance. At the genus level, the dominant genus of NDF and ADF groups are *Lactobacillus*, *Clostridium*, *Streptococcus*, *unclassified p Firmicutes*, *Prevotella*, *unclassified f Lachnospiraceae*, *Bacteroides*, *Ruminococcus*, *Eubacterium*, and *Roseburia* (Figures 1C,D). And the dominant species (mean relative abundance > 1%) in NDF group and ADF group were similar, mainly including *unclassified g Lactobacillus*, *Lactobacillus amylovorus*, *Lactobacillus reuteri*, *Clostridium* sp. *CAG:221*, *Streptococcus gallolyticus*, *Prevotella* sp. *CAG:279*, and *Firmicutes bacterium CAG:110* (Figures 1E,F). We found that the relative abundance of *Lactobacillus* in low digestibility groups of NDF and ADF was higher than that in high digestibility groups of NDF and ADF, and the *Lactobacillus* in L_NDF group was significantly higher than that in H_NDF group (*P* < 0.01; Figure 2).

### Screening the Gut Microbiota That Significantly Affect the Apparent Fiber Digestibility of Pig Using Linear Discriminant Analysis Effect Size Analysis

**FIGURE 3:**
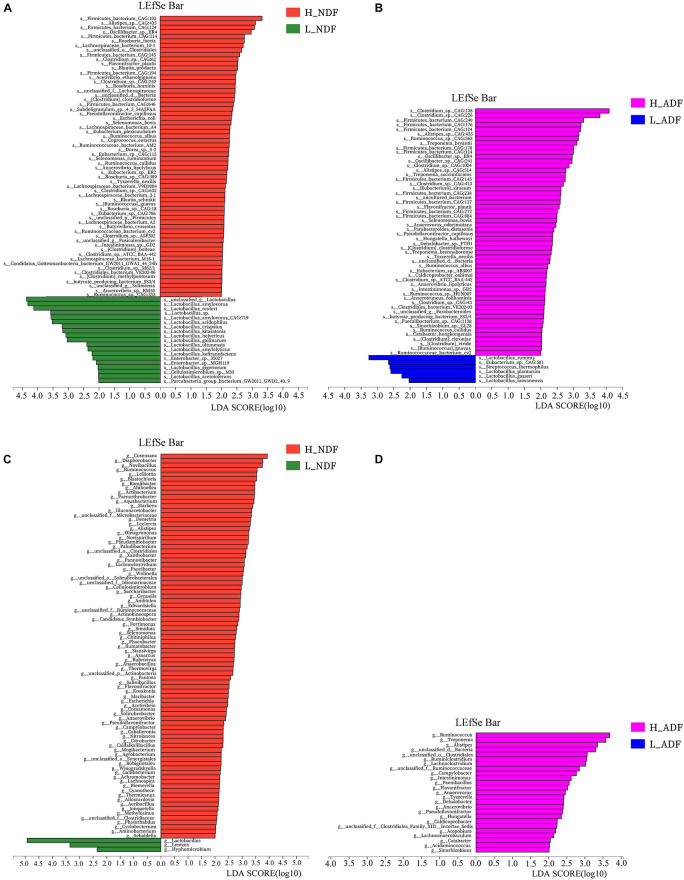
LEfSe analysis was performed to identify the species and genus in each sample that is closely contribute to fiber digestion with the greatest contribution. Significant species **(A,B)** and genus **(C,D)** identified by LEfSe were displayed (*P* < 0.05, LDA score > 2).

In order to find potential microorganisms that contribute to fiber degradation, we conducted linear discriminant analysis effect size (LEfSe) analysis. LEfSe determines the features most likely to explain differences between classes by coupling standard tests for statistical significance with additional tests encoding biological consistency and effect relevance. Firstly, non-parametric factorial Kruskal–Wallis sum-rank test was used to test the species or function abundance differences between different groups to obtain significantly different species or functions, then the influence of these different species or functions on the difference between groups was estimated by linear discriminant analysis (LDA) score. For NDF group, we found 62 bacterial biomarkers of species level may help to improve the apparent NDF digestibility in H_NDF group (LDA score > 2; Figure 3A). For ADF group, we also found 54 bacterial biomarkers of species level may help to improve the apparent ADF digestibility in H_ADF group (LDA score > 2; Figure 3B). Of note, many biomarkers belong to the *Clostridium*, *Ruminococcus*, *Eubacterium*, and *Roseburia*, and these genera contain many microorganisms with fiber degradation function. We observed that 17 bacterial biomarkers of species level in H_NDF and H_ADF groups were the same bacteria, such as *Firmicutes bacterium CAG:124*, *Alistipes* sp. *CAG:435*, *Firmicutes bacterium CAG:114*, *Oscillibacter* sp. *ER4*, *Firmicutes bacterium CAG:145*, *Flavonifractor plautii*, *Selenomonas bovis*, *Pseudoflavonifractor capillosus*, *Clostridium clostridioforme*, *Tyzzerella nexilis*, *R. albus*, *Clostridium* sp. *ATCC BAA-442*, *Anaerovibrio lipolyticus*, *Intestinimonas* sp. *GD2*, *butyrate-producing bacterium SS3/4*, *Ruminococcus callidus*, *Ruminococcus gnavus*, and *Ruminococcaceae bacterium cv2*. These shared some biomarkers, which may affect the apparent NDF digestibility and apparent ADF digestibility in pigs. In addition, *Roseburia faecis*, *Roseburia hominis*, *Selenomonas ruminantium*, and *Clostridium methylpentosum* in H_NDF group and *Treponema bryantii* in H_ADF group are bacteria that contribute to the degradation of non-starch polysaccharides in the intestinal tract of pigs. We also found that only L_NDF and L_ADF groups had significant enrichment of *Lactobacillus*, while H_NDF and H_ADF groups did not. At the genus level, we found 85 and 25 bacterial genera that may contribute to fiber degradation in H_NDF and H_ADF groups, respectively (LDA score > 2, Figures 3C,D). Where *g Ruminococcus*, *g Alistipes*, *g unclassified o Clostridiales*, *g Lachnoclostridium*, *g unclassified f Ruminococcaceae*, *g Campylobacter*, *g Flavonifractor*, *g Anaerovibrio*, and *g Pseudoflavonifractor* were common to H_NDF and H_ADF groups.

### Carbohydrate Active Enzyme Annotation Reveals Functional Differences Related to Fiber Decomposition of the Extreme High and Low Apparent Fiber Digestibility Groups

**FIGURE 4:**
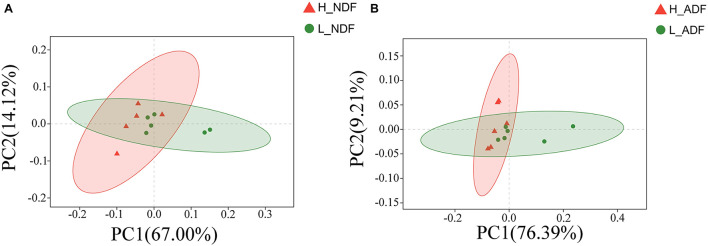
The principal coordinates analysis (PCoA) of CAZyme gene between high and low apparent fiber digestibility. PCoA plot show the significant difference in CAZyme gene diversity between H_NDF and L_NDF groups (**A**, *P* = 0.044), and H_ADF and L_ADF groups (**B**, *P* = 0.020).

We compared the functional differences of pig gut microbiota between H_NDF and L_ADF groups, H_ADF and L_ADF groups through functional annotation of metagenome with the carbohydrate active enzyme (CAZy) database. CAZyme database contains 6 classes of CAZy: GHs (glycoside hydrolases), GTs (glycosyltransferases), PLs (polysaccharide lyases), CBM (carbohydrate binding modules), CEs (carbohydrate esterases), and AAs (auxiliary activities). Through principal coordinates analysis (PCoA), we found that there were significant differences in CAZyme gene between H_NDF and L_NDF groups (*PERMANOVA*, *P* = 0.044, Figure 4A) and between H_ADF and L_ADF groups (*PERMANOVA*, *P* = 0.020, Figure 4B).

**FIGURE 5:**
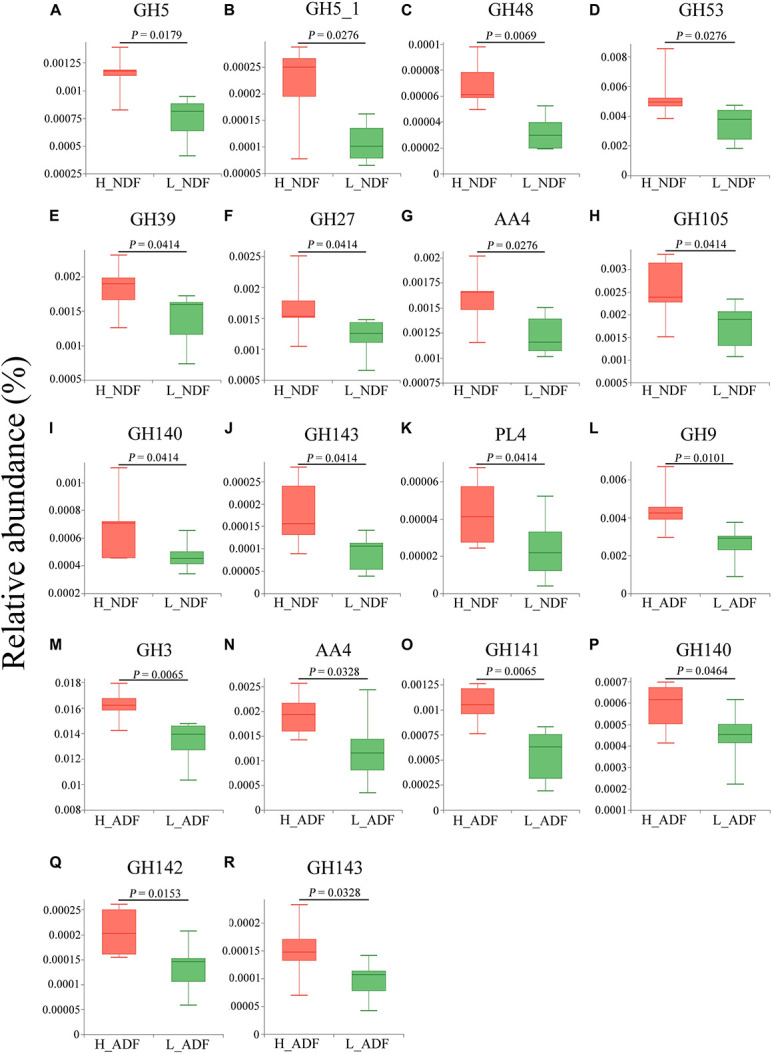
Tested the difference of CAZy families between H_NDF and L_NDF groups and H_ADF and L_ADF groups by Wilcoxon rank-sum test: **(A–C)** show the CAZy families were associated with the degradation of cellulose, **(D–F)** show the CAZy families were associated with the degradation of hemicellulose, **(G)** shows the CAZy families was associated with the degradation of lignin, **(H–K)** show the CAZy families were associated with the degradation of pectin. Tested the difference of CAZy families between H_ADF group and L_ADF group by Wilcoxon rank-sum test: **(L,M)** show the CAZy families were associated with the degradation of cellulose, **(N)** show the CAZy families was associated with the degradation of lignin, **(O–R)** show the CAZy families were associated with the degradation of pectin.

**FIGURE 6:**
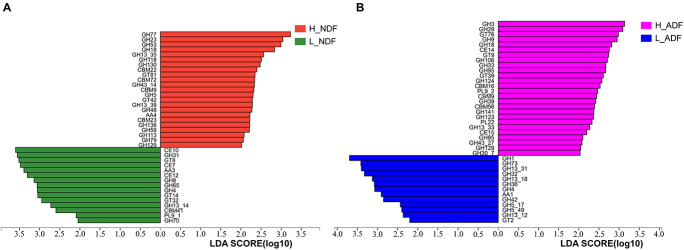
LEfSe analysis of CAZymes between H_NDF and L_NDF groups **(A)** and H_ADF and L_ADF groups **(B)**, respectively. CAZy families with a linear discriminant analysis (LDA) score > 2 were plotted.

In H_NDF group, GH5, GH5_1, and GH48 families contain many cellulose degrading enzymes, GH53, GH39, and GH27 families contain many enzymes that can participate in the degradation of hemicellulose, AA4 enzymes help lignin degradation, GH105, GH140, GH143, and PL4 families are associated with pectin degradation, and their relative abundance is significantly higher than that in L_NDF group (Figures 5A–K). Similarly in the H_ADF group, GH9 and GH3 families contain many cellulose degrading enzymes, AA4 enzymes help lignin degradation, GH141, GH140, GH142, and GH143 families are associated with pectin degradation, and their relative abundance is also significantly higher than that in L_ADF group (Figures 5L–R). Through LEfSe analysis, we further confirmed that GH5, GH48, GH53, GH39, and AA4 families in H_NDF group, GH9 and GH3 families in H_ADF group are important enzyme families closely associated with digestibility variation of pigs (Figure 6).

**FIGURE 7:**
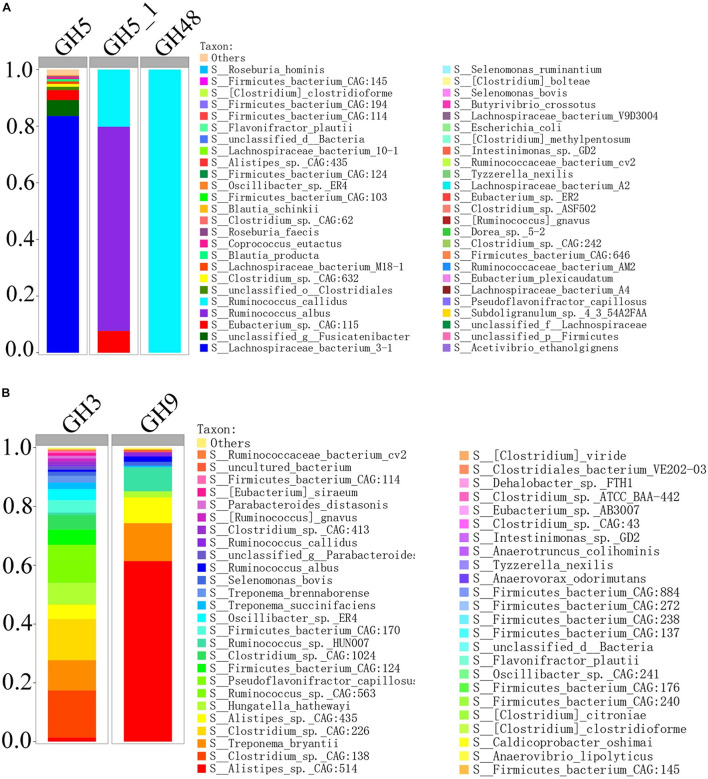
Percent contributions of GH5, GH5_1, and GH48 **(A)** and GH3 and GH9 **(B)** from 62 bacterial biomarkers of H_NDF group and 54 bacterial biomarkers of H_ADF group.

In order to compare the gene relative abundance ratio of cellulase among different biomarkers, we performed functional contribution analysis for these potential biomarkers in H_NDF and H_ADF groups. Among these biomarkers of H_NDF group, *Lachnospiraceae bacterium 3-1* (83.59%), *unclassified g Fusicatenibacter* (5.66%), *Eubacterium* sp. *CAG:115* (3.5%), *unclassified o Clostridiales* (1.17%), and *Clostridium* sp. *CAG:632* (1.01%) were the main bacteria with high relative abundance of GH5 family, and only *R. albus* (72.11%), *R. callidus* (20.19%), and *E.* sp. *CAG:115* (7.7%) contained GH5_1 family, and only *R. callidus* (100%) contained GH48 family (Figure 7A). Similarly, in these biomarkers of H_ADF group, *Clostridium* sp. *CAG:138* (16.04%), *Clostridium* sp. *CAG:226* (14%), *T. bryantii* (10.32%), *Ruminococcus* sp. *CAG:563* (7.49%), *Hungatella hathewayi* (7.4%), *P. capillosus* (5.45%), *Firmicutes bacterium CAG:124* (5.15%), *A.* sp. *CAG:435* (4.94%), *Clostridium* sp. *CAG:1024* (4.92%), *Firmicutes bacterium CAG:170* (4.17%), *O.* sp. *ER4* (3.12%), *Treponema succinifaciens* (2.31%), *Treponema brennaborense* (2.26%), *S. bovis* (1.36%), *A.* sp. *CAG:514* (1.32%), *Clostridium* sp. *CAG:413* (1.06%), *[Ruminococcus] gnavus* (1.02%), *R. albus* (0.73%), and *R. callidus* (0.55%) were the main bacteria with high relative abundance of GH3 family, and *A.* sp. *CAG:514* (61.39%), *T. bryantii* (12.91%), *A.* sp. *CAG:435* (8.78%), *Ruminococcus* sp. *HUN00* (8.18%), *H. hathewayi* (2.07%), *R. albus* (1.83%), *R. callidus* (1.45%), and *S. bovis* (1.34%) were the main bacteria with high relative abundance of GH9 family (Figure 7B). In general, these bacteria have abundant genes related to cellulose degradation. We speculated that these bacteria can promote the degradation of fiber and improve the apparent fiber digestibility of pigs.

### KEGG Annotation Reveals Functions Differences Related to Fiber Decomposition of the Extreme High and Low Apparent Fiber Digestibility Groups

**FIGURE 8:**
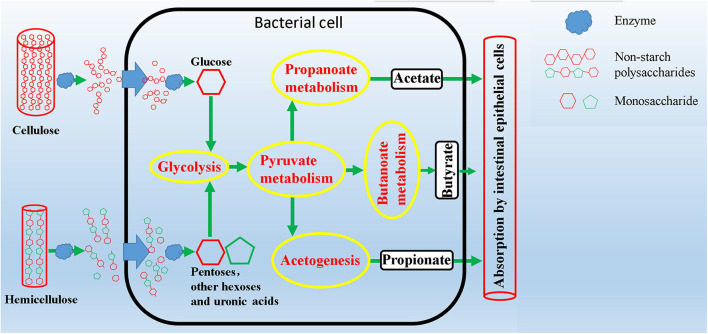
Schematic overview of intestinal microbial metabolic pathways from degradation of complex polysaccharides to production of short chain fatty acids.

**FIGURE 9:**
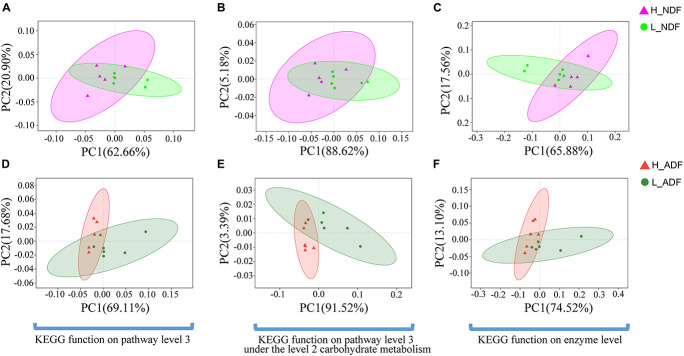
The principal coordinates analysis (PCoA) shows the significant difference in KEGG function diversity between H_NDF and L_NDF groups and between H_ADF and L_ADF groups. **(A)** (*PERMANOVA*, *P* = 0.048) shows the significant difference in the KEGG function on pathway level 3 of H_NDF and L_NDF groups. **(B)** (*PERMANOVA*, *P* = 0.053) shows the difference in the KEGG function on pathway level 3 under the level 2 carbohydrate metabolism of H_NDF and L_NDF groups. **(C)** (*PERMANOVA*, *P* = 0.033) shows the significant difference in the KEGG function on enzyme level of H_NDF and L_NDF groups. **(D)** (*PERMANOVA*, *P* = 0.006) shows the significant difference in the KEGG function on pathway level 3 of H_ADF and L_ADF groups. **(E)** (*PERMANOVA*, *P* = 0.006) shows the significant difference in the KEGG function on pathway level 3 under the level 2 carbohydrate metabolism of H_ADF and L_ADF groups. **(F)** (*PERMANOVA*, *P* = 0.021) shows the significant difference in the KEGG function on enzyme level of H_ADF and L_ADF groups.

**FIGURE 10:**
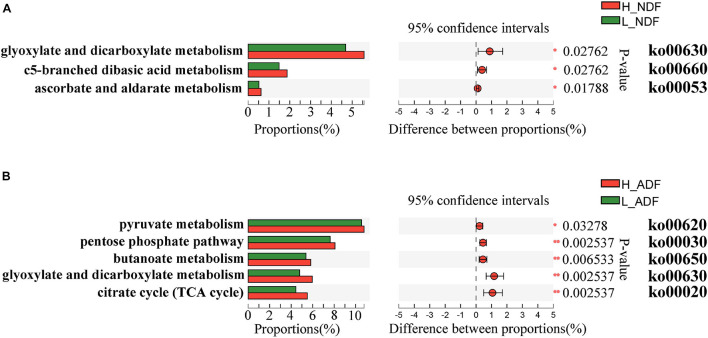
The KEGG on pathway level 3 under the level 2 carbohydrate metabolism functional differences were analyzed between H_NDF group and L_NDF group **(A)** and between H_ADF group and L_ADF group **(B)** by Wilcoxon rank-sum test. An asterisk (*P* < 0.05) or double asterisk (*P* < 0.01) indicate significantly different.

Plant complex polysaccharides such as cellulose and hemicellulose in feed will be converted into short-chain fatty acids (SCFAs) through the metabolic process shown in Figure 8, and the SCFAs were absorbed and utilized by posterior intestinal epithelial cells. We compared the functional capacity of the intestinal microflora between high and low digestibility groups of NDF and ADF through the functional annotation of metagenome with the KEGG database. We found that there were significant differences in KEGG function of gut bacterial communities between the high and low apparent fiber digestibility groups of NDF group and ADF group (Figure 9). For these important metabolisms in Figure 8, the functional relative abundance of glyoxylate and dicarboxylate metabolism (ko00630), c5-branched dibasic acid metabolism (ko00660), and ascorbate and aldarate metabolism (ko00053) in H_NDF group were significantly higher than that in L_NDF group (Figure 10A), and the functional relative abundance of pyruvate metabolism (ko00620), pentose phosphate pathway (ko00030), butanoate metabolism (ko00650), glyoxylate and dicarboxylate metabolism (ko00630), and citrate cycle (TCA cycle; ko00020) in H_ADF group were significantly higher than that in L_ADF group (Figure 10B). We found that in the NDF group and the ADF group, the functional relative abundance of glyoxylate and dicarboxylate metabolism (ko00630) was both significantly higher in the high apparent fiber digestibility group than that in the low apparent fiber digestibility group. Among the pathways with significant differences, pyruvate metabolism (ko00620) and butanoate metabolism (ko00650) are both important functional pathways for fiber digestion into SCFA.

## Discussion

In the study of the key bacterial species and biological function related to the apparent fiber digestibility of Suhuai pigs, shotgun metagenomic sequencing has shown some advantages compared to 16S rRNA gene sequencing. Metagenome sequencing can further understand the relationship between genes and bacterial function. Our results indicated that the intestinal microflora of Suhuai pigs with high apparent fiber digestibility has more potential genes or functions that contribute to fiber degradation.

There are a large number of microorganisms in animal intestines, and their rich genes can make up for the lack of congenital function of animals, just like an organ of the body. The results show that Firmicutes and Bacteroides are dominant and their sum of relative abundance reached more than 90% in the intestine of Suhuai pigs (Figure 1), which is consistent with the research of Adhikari et al. (2019) on PIC genetics nursery pigs and the research of Niu et al. (2019, 2020) on Sutai pigs and Suhuai pigs. Previous studies have found that Firmicutes and Bacteroides were capable of degrading many carbohydrate polymers in different ruminants, including cattle (Stewart et al., 2018, 2019; Gharechahi et al., 2021), camels (Gharechahi and Salekdeh, 2018), and moose (Svartström et al., 2017). The proportion of Firmicutes in the intestinal tract of pigs with low digestibility (L_NDF: 85.85%; L_ADF: 86.31%) was higher than that of high digestibility (H_NDF: 79.88; H_ADF: 78.76%) and the proportion of Bacteroidetes in the intestinal tract of pigs with high digestibility (H_NDF: 12.14%; H_ADF: 12.08%) was higher than that of low digestibility (L_NDF: 7.16%; L_ADF: 6.89%), which indicated that the community competition between Firmicutes and Bacteroidetes may be related to the fiber degradation ability of pigs (Figure 1). A previous study shown that the results indicated that the increase of apparent fiber digestibility of pig may be closely associated with the increase of relative abundance of intestinal Bacteroides (Gharechahi et al., 2021). Bacteroides are the most abundant microbes in rumen. They are rich in CAZyme gene and can degrade the main components of plant cell wall, such as xyloglucans, glucuronylxylans, and pectin. The proportion of Firmicutes and Bacteroidetes in pig intestinal tract will affect the ability of pig to degrade fiber. In this study, the top three bacteria species with relative abundance belong to the genus *Lactobacillus* (Supplementary Figure 1). In NDF and ADF groups, the content of genus *Lactobacillus* in the samples with low apparent fiber digestibility (L_NDF: 32.02%; L_ADF: 34.24%) was higher than that in the samples with high apparent fiber digestibility (H_NDF: 14.24%; H_ADF: 18.50%; Figure 1). Ivarsson (2012) found that wheat bran diets stimulate the growth of *Lactobacillus* in the ileal microflora of pigs. In the research of Molist et al. (2009), it was also found that wheat bran diet increased the abundance of *Lactobacillus*, but the results were not significant. In this experiment, the content of wheat bran in the diet was 24%, suggesting that that wheat bran may be promoted the proliferation of *Lactobacillus* in the intestine of Suhuai pigs. The higher content of *Lactobacillus* in low digestibility group may affect the colonization of other microorganisms that can participate in fiber degradation. As we all know, cellulose, hemicellulose and lignin are important components of plant cell walls. This study found that bacteria in the H_NDF and H_ADF groups contained a large number of cell wall degrading enzyme genes of CAZyme families, and their abundance was significantly higher than that of L_NDF and L_ADF groups. GH5, GH5_1, GH27, GH39, GH48, GH53, GH105, GH140, GH143, AA4, and PL4 are significantly more abundant CAZyme family that contributes to the degradation of fiber in H_NDF group than that in L_NDF group. GH3, GH9, GH141, GH142, and AA4 are significantly more abundant CAZyme family that contributes to the degradation of fiber in H_ADF group than that in L_ADF group (Figure 5). Many studies have shown that adding cell wall degrading enzymes will speed up the removal of fermentative substrates such as starch in the animal’s intestines, leading to changes in the structure of intestinal microbes, thereby reducing the abundance of *Lactobacillus* in the intestines (Hübener et al., 2002; Jia et al., 2009; Bedford and Cowieson, 2012). We speculated that a large number of cell wall degrading enzymes secreted by intestinal microbes of pigs with high apparent fiber digestibility affect the structure of the gut microbiota, resulting in low abundance of *Lactobacillus* in group with high apparent fiber digestibility.

In order to understand the difference of the intestinal microbes between high and low apparent fiber digestibility in Suhuai pigs, we used LEfSe to identify the significantly enriched potential biomarkers related to apparent fiber digestibility in H_NDF and H_ADF groups. LEfSe is an algorithm that focuses on statistical significance and biological consistency (Segata et al., 2011). In this study, we found 62 key bacteria, namely biomarkers, that were significantly associated with apparent fiber digestibility in H_NDF group and 47 in H_ADF group (Figure 3). We also found that most of these key bacteria belong to the *Clostridium*, *Ruminococcus*, *Eubacterium*, and *Roseburia*, which found to be associated with the production of SCFA in the intestine (Pryde et al., 2002; Louis and Flint, 2009; Kumari, 2013; Jiang et al., 2017). *Clostridium* is one of the largest bacterial genus (Dong et al., 2010), which has multi-enzyme system involving cellulosome and xylanosome (Thomas et al., 2014), and some of its members have the ability to degrade the complex cellulosic polymers and various cellulosic degradation by-products (Varel et al., 1989; Tracy et al., 2012). *Ruminococcus* is a bacterial genera commonly found in the rumen of ruminants and plays an important role in the fiber fermentation of animal hindgut (Blaut, 2011; Kim et al., 2011; Walker et al., 2011). *C.* sp. *CAG:138*, *C.* sp. *CAG:226*, *C.* sp. *CAG:413*, *C.* sp. *CAG:1024*, and *C.* sp. *CAG:632* of *Clostridium* and *R.* sp. *CAG:563* and *R.* sp. *HUN00* of *Ruminococcus* have not been reported to be associated with fiber digestion of pig. *Roseburia* is a typical butyrate producing bacteria. A previous study has shown that *R. faecis* could degrade oligofructose and produce butyrate (Falony et al., 2009). A recent study found that rats fed with fresh *R. hominis* cell suspension could increase the concentration of butyrate in their cecum (Zhang et al., 2018). A total of 3 biomarkers were identified to belong to genus *Subdoligranulum*. *Subdoligranulum*, which is a kind of short chain fatty acid-producing bacteria and it has been reported in some literature that *Subdoligranulum* sp. with probiotic potential and contribute to the digestion of dietary fiber and the production of SCFA (Zheng et al., 2019). It is worth noting that *Selenomonas ruminantium* is the dominant bacterium in rumen that can secrete beta-D-xylosidase, which has the potential to be used in saccharification of lignocellulosic biomass for producing biofuels and can participate in the digestion of fiber in rumen (Jordan and Wagschal, 2010; Sawanon et al., 2011). Li et al. (2017) studied the effect of diet on intestinal microbial community, and they found a correlation between *Butyrivibrio crossotus* and xylanase/xylosidase. In 1989, one study (Himelbloom and Canale-Parola, 1989) isolated a new species of *Clostridium* and proposed a new species named *C. methylpentosum*. It can produce two methylpentoses (L-rhamnose, L-fucose) and two pentoses (L-lyxose and D-arabinose) by fermentation in intestine and may participate in the digestion of pectin in the intestine. *Treponema* spp. is a common type of bacteria helps in the degradation of soluble fibers in rumen (Bekele et al., 2011). A previous study have shown that plant polysaccharides could be degraded by *Treponema* strains of rumen (Ziołecki, 1979). In the study of Quan et al. (2020), they found that *T. bryantii*, which was enriched in H_ADF group of this study, is potentially associated with higher feed efficiency of pigs through LEfSe analysis. Some studies have also shown that there is a beneficial interaction between *T. bryantii* with the cellulolytic bacterium *F. succinogenes* (Stanton and Canale-Parola, 1980). Compared with other biomarkers, we found that *T. bryantii* has a great contribution to multiple cellulase-related families. In the potential biomarkers of H_ADF group, the relative contribution to GH3 and GH9 of *T. bryantii* reaches 10.32 and 12.91%. These results indicate that *T. bryantii* has a great potential to degrade fiber of diet. Among the share biomarkers enriched in H_NDF group and H_ADF group, *R. albus*is an important member of rumen community and the main fiber degrading bacteria in rumen, which can produce a lot of cellulase and hemicellulase (Forsberg et al., 1997; Wina et al., 2006). It plays an important role in the degradation of plant cell wall and can hydrolyze cellulose non-cellulosic structural polysaccharides (Leatherwood, 1965; Grinberg et al., 2015). In this study, we also found that the relative contribution to GH5_1 of *R. albus* reaches 72.11% and only *R. callidus* contains GH48 family in all potential bacterial biomarkers of H_NDF group. *R. callidus* is a producer of short-chain fatty acids (SCFAs), which can be used as a bacterial biomarker for improving the health and production performance of meat rabbits (Ye et al., 2021). *S. bovis* is a kind of separable bacteria existing in the rumen contents of yak, which can utilize cellobiose, arabinose, mannose, glucose, etc. (Zhang and Dong, 2009). Interestingly, *A. lipolyticus* was considered to be one of the major species participating in lipid hydrolysis in ruminant animals (Edwards et al., 2013; Privé et al., 2013) and is enriched in both H_NDF group and H_ADF group. We located several representative bacterial biomarkers in the intestines of Suhuai pigs. They generally exist in rumen of ruminants and play a positive role in the digestion of dietary fiber, such as *S. ruminantium* and *R. albus*, etc. In conclusion, we speculate that the extremely high apparent fiber digestibility of Suhuai pigs is associated with a large number of microorganisms with the function of degrading fiber in the intestine. Meanwhile, we found 85 genera associated with fiber digestion in H_NDF group (LDA score > 2; Figure 3C), for ADF group, we also found 25 genera associated with fiber digestion in H_ADF group (LDA score > 2; Figure 3D). These results confirmed the above results and indicated that many important genera may be associated with the process of fiber digestion in the intestine of Suhuai pigs.

Our results showed that the difference in the function of the gut microbiota is likely to be the main reason for the variation of the apparent fiber digestibility of Suhuai pigs (Figures 4, 9). We have found the gene abundance of many families that can degrade cellulose and hemicellulose and important KEGG pathways related to fiber degradation were significantly higher in the group with high apparent digestibility than in the group with low apparent digestibility. We retrieved the literature and found that microbial cellulases fall into GH families 3, 5, 9, 48, etc. (Pope et al., 2010; Zhu et al., 2011; Lombard et al., 2014; Romero Victorica et al., 2020). Our results showed that the relative abundance of GH5, GH5_1, and GH48 families in the H_NDF group was significantly higher than that in the L_NDF group, and the relative abundance of GH3 and GH9 families in the H_ADF group was significantly higher than that in the L_ADF group. In addition, in NDF and ADF groups, the relative abundance of many families was associated with hemicellulose and pectin degradation in high apparent digestibility group is significantly higher than that in the low apparent digestibility group. In the H_NDF group, GH53, GH39, and GH27 families are families that are significantly enriched and contains many enzymes that can participate in the degradation of hemicellulose (Zhao et al., 2013; Coconi Linares et al., 2020; Romero Victorica et al., 2020), while GH105, GH140, GH143, and PL4 families are significantly enriched and contains many enzymes that can participate in the degradation of pectin (Zhao et al., 2013; Ndeh et al., 2017). And in the H_ADF group, GH140, GH142, and GH143 families are significantly enriched and contain many enzymes that can participate in the degradation of pectin (Ndeh et al., 2017). The mechanism of lignin degradation is still unclear, and the structural complexity, insolubility and high molecular weight of lignin make its enzymatic degradation challenging for microorganisms to obtain and convert polysaccharides. AA4 enzyme is active to many aromatic compounds produced in the process of lignin degradation, and plays a certain role in lignin degradation (Levasseur et al., 2013). Our results showed that AA4 family was significantly enriched in H_NDF group and H_ADF group. The fiber is first decomposed by enzymes secreted by intestinal bacteria, and then the products enter the bacteria for further decomposition and digestion to produce SCFA. In the NDF and ADF groups, we found that the relative abundance of many important KEGG pathways related to fiber degradation was significantly higher in the high apparent digestibility group than in the low apparent digestibility group, such as dicarboxylate metabolism (ko00630) in H_NDF group and pyruvate metabolism (ko00620) and butanoate metabolism (ko00650) in H_ADF group (Tanca et al., 2017). We believed that these differences in enzyme families and functional pathways related to fiber digestion can provide a reasonable explanation for the variation of apparent fiber digestibility among individuals in Suhuai pigs.

By analyzing the functional differences of intestinal microbial composition and function in Suhuai pigs, we found that there were significant differences in the microorganisms related to fiber degradation and gene families with cellulose, hemicellulose, pectin, or lignin degrading activity between H_NDF and L_NDF groups and H_ADF and L_ADF groups. The important microorganisms relate to fiber degradation are mainly *R. faecis*, *R. hominis*, *S. ruminantium*, *Butyrivibriocrossotus*, and *C. methylpentosum* in H_NDF group, *T. bryantii* in H_ADF group, and *R. albus*, *R. callidus*, and *S. bovis* were shared in both H_NDF and H_ADF groups. These microorganisms have been confirmed by researchers through *in vitro* experiments that they have a certain role in the process of fiber degradation. Compared with other biomarkers in H_NDF group, the relative abundance of GH5 in *Lachnospiraceae bacterium 3-1* was significantly higher. And compared with other biomarkers in H_ADF group, the relative abundance of GH9 in *A.* sp. *CAG:514* was significantly higher (Figure 7). Among the biomarkers of H_NDF group, only three bacteria have GH5_1 family gene, *E.* sp. *CAG:115* is one of them. We speculate that *Lachnospiraceae bacterium 3-1*, and *A.* sp. *CAG:514* are likely to have the ability to degrade fiber, but there is no report so far. GH3, GH5, GH9, and GH48 of the gene families with cellulose degrading activity in high apparent digestibility group is significantly more abundant than those in low apparent digestibility group. And important pathways are associated with complex polysaccharide such as dicarboxylate metabolism (ko00630), pyruvate metabolism (ko00620), and butanoate metabolism (ko00650) are enriched in high apparent digestibility group. We speculated that the key microorganisms and the gene families with fiber degrading activity caused the different apparent fiber digestibility of Suhuai pigs.

Taken together, we found that gut microbial community structure and functional components have a significant impact on the fiber digestibility of pigs. Fully understanding gut microbiota that can improve fiber digestibility of pig can promote the application of coproducts in pig feed, alleviate the shortage of feed raw materials, and promote the sustainable development of pig industry.

## Conclusion

We found that the proportion of Firmicutes and Bacteroidetes in pig intestinal tract was inconsistent between high and low apparent fiber digestibility groups. Further study is necessary to judge whether the abundance of these bacteria affects the fiber digestibility. A total of62 microbial species in H_NDF group and 54 microbial species in H_ADF group may be related to fiber digestion of pigs were identified, of which there are also many microbial species that have been reported to participate in the process of fiber digestion. *Lachnospiraceae bacterium 3-1* and *A.* sp. *CAG:514* may have strong fiber degradation ability, but it has not been reported, which needs further study. Many gene families with cellulose, hemicellulose, pectin, and lignin degrading activity such as GH3, GH5, GH39, GH48, GH53, and AA4 families and some important pathways are associated with complex polysaccharide metabolism such as glyoxylate and dicarboxylate metabolism (ko00630), pyruvate metabolism (ko00620), and butanoate metabolism (ko00650) in the high digestibility group may be associated with the variation of apparent fiber digestibility in pigs. In conclusion, Suhuai pigs with high apparent fiber digestibility contained more abundant genes and bacteria related to fiber digestion, which led to their strong fiber digestion potential.

## Data Availability Statement

The datasets presented in this study can be found in online repositories. The names of the repository/repositories and accession number(s) can be found below: https://www.ncbi.nlm.nih.gov/, PRJNA735412.

## Ethics Statement

The animal study was reviewed and approved by the Institutional Animal Welfare and Ethics Committee of Nanjing Agricultural University, Nanjing, China [Certification No: SYXK (Su) 2017 - 0007].

## Author Contributions

RH and PL contributed to conception and design of the study. GL, QN, GP, BW, TD, PN, and QL collected and assembled the data. GL performed the statistical analysis and wrote the first draft of the manuscript. RH, PL, and LH wrote sections of the manuscript. RH, PL, LH, and SK contributed to the revision of the manuscript. All authors have read and agreed to the published version of the manuscript.

## Conflict of Interest

QL was employed by company Huaiyin Xinhuai Pig Breeding Farm. The remaining authors declare that the research was conducted in the absence of any commercial or financial relationships that could be construed as a potential conflict of interest.

## Publisher’s Note

All claims expressed in this article are solely those of the authors and do not necessarily represent those of their affiliated organizations, or those of the publisher, the editors and the reviewers. Any product that may be evaluated in this article, or claim that may be made by its manufacturer, is not guaranteed or endorsed by the publisher.

## Footnotes

1
www.illumina.com

2
http://bio-bwa.sourceforge.net

3
https://github.com/voutcn/megahit

4
http://www.bioconductor.org/packages/release/bioc/html/metagene.html

5
http://www.ncbi.nlm.nih.gov/Taxonomy/taxonomyhome.html/index.cgi?chapter=tgencodes#SG1

6
http://www.bioinformatics.org/cd-hit/

7
https://help.rc.ufl.edu/doc/SOAPaligner

8
http://www.genome.jp/kegg/

9
http://blast.ncbi.nlm.nih.gov/Blast.cgi

10
http://www.cazy.org/

11
http://www.hmmer.org/
