# Supplementary material for: Metagenomic Analysis Reveals New Microbiota Related to Fiber Digestion in Pigs
Source: Front Microbiol. 2021 Nov 18;12:746717. doi: 10.3389/fmicb.2021.746717 (PMC8637618; doi:10.3389/fmicb.2021.746717)
Supplement: Supplementary file 1 [file Data_Sheet_1.docx]

Supplementary Material

# Supplementary Figures and Tables

## Supplementary Tables

# Supplementary Table 1. Description of the assembly results of fecal microbes from Suhuai pigs with high and low apparent digestibility groups in NDF and ADF groups.

| Group | Raw reads | Clean reads | Percent in raw reads (%) | Contigs | ORFs |
| --- | --- | --- | --- | --- | --- |
| H_NDF | 95915978.40 ± 4790352.24 | 95171251.60 ± 4892749.02 | 99.22 ± 0.18 | 845737.80 ± 153702.90 | 1369863.00 ± 230574.24 |
| L_NDF | 93707055.33 ± 5365275.65 | 92966931.00 ± 5317605.49 | 99.21 ± 0.10 | 730093.67 ± 133207.43 | 1178173.33 ± 203774.49 |
| H_ADF | 96811367.67 ± 3955192.31 | 95925215.67 ± 3998766.43 | 99.08 ± 0.23 | 788965.33 ± 119010.75 | 1307673.00 ± 177444.97 |
| L_ADF | 90139704.00 ± 2424608.25 | 89453259.00 ± 2455243.03 | 99.24 ± 0.08 | 740542.00 ± 80899.85 | 1096034.00 ± 247157.50 |

Note: The form of data is mean ± SD

## Supplementary Figures

# Supplementary Figure 1. The microbiota composition of each fecal sample at species level in NDF (A) and ADF (B) groups and for those less than 0.5% were merged into others for display.

|  |  |
| --- | --- |

# Supplementary Figure 2. Non-metric multidimensional scaling (NMDS) (A) and Principal coordinate analysis (PCoA) (B) profile of gut microbial diversity at species level between the H_NDF and L_NDF groups using a Bray-Curtis metric.
